# Early Cardiac Responses to Half-Marathon Running in Amateur Athletes: Implications for Cardiovascular Health and Safe Exercise Participation

**DOI:** 10.3390/jfmk11020211

**Published:** 2026-05-27

**Authors:** Kyriakos-Marios Oikonomou, Apostolia Ntovoli, Nikolaos Koutlianos, Maria Anifanti, Christos Mantzios, Sotiria Iliopoulou, Anastasia Mata, Ilias Marios Oikonomou, Kostas Alexandris, Evangelia Kouidi

**Affiliations:** 1 Laboratory of Sports Medicine, Department of Physical Education and Sports Science, Aristotle University of Thessaloniki, 57001 Thessaloniki, Greece; antovoli@phed.auth.gr (A.N.); koutlian@phed.auth.gr (N.K.); manyfant@phed.auth.gr (M.A.); kalexand@phed.auth.gr (K.A.); kouidi@phed.auth.gr (E.K.); 2 Cardiology Department, General Hospital of Giannitsa, 58100 Giannitsa, Greece; amata@gng.gr; 3 Department of Life and Health Sciences, Frederick University, 1036 Nicosia, Cyprus; 4 Cardiology Department, University General Hospital of Thessaloniki “AHEPA”, 54636 Thessaloniki, Greece; chrismantzios1994@gmail.com; 5 Cardiology Department, General Hospital of Thessaloniki “Papanikolaou”, 57010 Thessaloniki, Greece; sotiria.ili26@gmail.com; 6 Department of Transplant Surgery, Aristotle University of Thessaloniki, 54642 Thessaloniki, Greece; i.m.oikonomou@gmail.com

**Keywords:** cardiovascular health, exercise participation, amateur runners, half-marathon, diastolic indices, echocardiography, cardiac biomarkers, hs-cTnI

## Abstract

**Background**: Recreational half-marathon participation is increasing, particularly among middle-aged amateur runners, yet the interpretation of early post-race cardiac findings remains challenging in exercise-based cardiovascular health evaluation. This exploratory study assessed early post-race changes in left ventricular diastolic indices and circulating biomarkers in 20 healthy amateur runners (80% male; mean age 50.7 ± 12.3 years) after the 11th PELLA HALF MARATHON (21.1 km). **Methods**: Participants underwent transthoracic echocardiography and venous blood sampling within 30 days before the race and within 30 min after finishing. Diastolic assessment included the E/A ratio, tissue Doppler early diastolic myocardial velocity (e′), the E/e′ ratio, isovolumic relaxation time (IVRT), and left atrial area. Biomarkers included C-reactive protein (CRP), creatine phosphokinase (CPK), creatine kinase-MB (CK-MB), and high-sensitivity cardiac troponin I (hs-cTnI). **Results**: Post-race assessment showed a consistent pattern of lower early diastolic filling/relaxation indices, higher IVRT and left atrial area, and significant increases in all measured biomarkers. hs-cTnI exceeded the sex-specific 99th percentile upper reference limit in 7/20 participants (35%). **Conclusions**: Half-marathon completion was associated with early echocardiographic and biomarker changes in this cohort of amateur runners. These findings are consistent with acute physiological cardiac stress and may help clinicians contextualise early post-race abnormalities when advising on vigorous endurance exercise participation. However, subclinical myocardial injury cannot be excluded without serial biomarker assessment and advanced imaging, and the findings should be interpreted as exploratory because of the small convenience sample, absence of a control group, lack of hydration assessment, and single early post-race timepoint.

## 1. Introduction

Participation in recreational endurance running continues to expand worldwide, and half-marathon events are especially popular among middle-aged adults seeking cardiovascular fitness and long-term health benefits through structured exercise. In this context, understanding the acute cardiac response to vigorous endurance participation is relevant not only to sports cardiology but also to exercise counselling, cardiovascular risk communication, and the safe promotion of physical activity in community-based populations [1,2,3,4,5,6].

Regular exercise confers clear cardiovascular benefits, but prolonged endurance events impose acute haemodynamic, metabolic, and inflammatory stress distinct from habitual training loads. The half-marathon is particularly relevant because it is widely accessible to non-elite athletes, attracts a heterogeneous amateur population, and can provoke measurable cardiovascular responses without the more prolonged physiological burden often associated with full marathon running. For clinicians and exercise professionals, distinguishing expected short-term post-race responses from findings that may warrant follow-up is important when supporting safe exercise participation [1,2,3,4,7,8,9].

Left ventricular diastolic indices are particularly sensitive to acute changes in preload, heart rate, and myocardial relaxation. In this study, the principal indices of interest were the transmitral early-to-late filling ratio (E/A), tissue Doppler early diastolic myocardial velocity (e′) as an index of myocardial relaxation, the E/e′ ratio as a surrogate of filling pressure, isovolumic relaxation time (IVRT), and left atrial (LA) area. Commonly reported post-exercise findings include lower E/A ratio and e′ velocity together with longer IVRT; however, these measurements may reflect both physiological loading changes and true transient impairment of relaxation. Accordingly, isolated post-race echocardiographic changes should be interpreted cautiously and in the context of biomarker kinetics and study design [2,3,4,10].

Biomarker elevations after endurance exercise, including hs-cTnI, CK-MB, CPK, and CRP, are also well described. Potential mechanisms linking diastolic perturbation and biomarker release include tachycardia-related shortening of filling time, preload reduction from vasodilation and dehydration, catecholamine-mediated wall stress, transient alterations in calcium handling, and increased membrane permeability during strenuous exercise. In many athletes, these increases appear to reflect physiological stress responses rather than overt myocardial necrosis; nevertheless, interpretation is challenging when measurements are obtained at only one post-exercise timepoint or when complementary imaging is unavailable. This distinction is especially important in real-world exercise promotion, where isolated post-race biomarkers or echocardiographic abnormalities may trigger unnecessary concern if interpreted without physiological context [7,8,9,11].

Despite this growing body of work, no prior study has combined a comprehensive echocardiographic assessment of diastolic function with a full cardiac and inflammatory biomarker panel (CRP, CPK, CK-MB, and high-sensitivity cardiac troponin I evaluated against sex-specific 99th percentile upper reference limits) in middle-aged amateur half-marathon runners assessed within 30 min of race completion. The present exploratory study aims to address this gap by characterising the early post-race cardiac response in this clinically relevant, real-world population and by examining preliminary associations between biomarker elevations and echocardiographic changes within the same individuals.

Although marathon and elite-athlete data are extensive, fewer studies have combined echocardiographic and biomarker assessment in amateur half-marathon runners, particularly in older recreational participants. This subgroup is clinically relevant because mildly abnormal post-race biomarkers or Doppler findings in middle-aged amateur runners may prompt medical evaluation despite the absence of symptoms. Better characterisation of these early responses may improve post-exercise clinical interpretation and support safer participation in vigorous physical activity [5,6,12,13,14,15,16,17,18,19,20].

The aim of the present exploratory observational study was to assess early post-race changes in left ventricular diastolic indices and cardiac biomarkers in amateur half-marathon runners, with potential relevance to cardiovascular health monitoring in recreational exercise settings. We hypothesised that race completion would be associated with reductions in E/A ratio and e′ velocity, prolongation of IVRT, and increases in circulating biomarkers, while recognising that the design does not support causal or mechanistic inference.

## 2. Materials and Methods

### 2.1. Study Design and Participants

This prospective single-centre observational study included 20 healthy amateur runners participating in the 11th certified PELLA HALF MARATHON. All participants completed the same race under comparable environmental and course conditions. Amateur runners were defined as non-professional endurance athletes with at least three years of active participation in long-distance running events (≥3 sessions/week), training 5–10 h per week, without structured elite-level coaching and without financial compensation for athletic performance [21]. For the purposes of this study, “healthy” was operationalised as the absence of known cardiovascular disease, recent systemic inflammation, relevant cardiometabolic medication use, abnormal screening blood pressure requiring exclusion, or structural heart disease on the pre-race assessment.

Inclusion criteria required regular training of more than three sessions per week, completion of the race within three hours, and no known cardiovascular disease, systemic inflammation within the preceding four weeks, or medication affecting cardiac or skeletal muscle metabolism. All participants provided written informed consent. The study was conducted in accordance with the Declaration of Helsinki and approved by the Ethics Committee of the School of Physical Education and Sport Science, Aristotle University of Thessaloniki (approval number 214/2024) and the Scientific Council of Giannitsa General Hospital (Protocol No. 583/16.01.2025). This operational definition does not exclude subclinical or exercise-related physiological variation, but it reduces the likelihood that overt pre-existing disease accounted for the observed post-race findings.

All participants were instructed to maintain their habitual training routine during the 30-day pre-race window and to avoid competitive events, intense interval training, and races within the seven days preceding the pre-race assessment. At the pre-race visit, each participant confirmed the absence of systemic infection (consistent with the inclusion criterion of no systemic inflammation within four weeks), no acute injuries, and no significant deviations from their established training programme. Detailed quantitative training-load monitoring during this window was not performed and is acknowledged as a limitation. The 11th PELLA HALF MARATHON took place in early September 2024 under typical late-summer conditions for the region (climatological norms for the Giannitsa station: morning temperature 17–22 °C, relative humidity 55–60%; data from the Hellenic National Meteorological Service). Formal recording of race-day environmental parameters was not part of the study protocol; all participants ran the same course under identical environmental exposure, eliminating between-subject environmental variability within the cohort.

No formal a priori sample size calculation was performed. The sample of *n* = 20 represents a convenience sample from a single registered athletic club and reflects the logistical constraints of immediate post-race assessment in a real-world field setting. The study was therefore designed as exploratory and hypothesis-generating rather than confirmatory. Of 27 initially approached, seven were excluded: one for antihypertensive medication use, one for elevated blood pressure at pre-race assessment, one for mild aortic regurgitation on echocardiography, and four who did not attend post-race assessment.

### 2.2. Echocardiographic Assessment

A comprehensive pre-race assessment was performed within 30 days before race participation at the Cardiology Department of Giannitsa General Hospital and included medical history, 12-lead resting ECG, and transthoracic echocardiography. Immediately after the race, and within 30 min of finishing, participants underwent repeat echocardiography and venous blood sampling at the same facility. The 30-min post-race window was selected to capture the earliest standardised post-exercise response while minimising confounding from participant movement, re-warming, substantial fluid intake, and prolonged recovery. This design captured early post-race changes but did not permit assessment of biomarker or echocardiographic recovery.

Standard two-dimensional transthoracic echocardiography was performed on a GE Vivid E9 system (GE HealthCare, Chicago, IL, USA) according to American Society of Echocardiography (ASE) and European Association of Cardiovascular Imaging (EACVI) recommendations [10]. All studies were acquired by a single experienced operator (K.M.O.). Measurements were averaged over three consecutive cardiac cycles in sinus rhythm. Offline analysis was performed by an independent cardiologist blinded to timepoint (pre-race vs. post-race) and reviewed by a second operator (C.M. or S.I.). Heart rate was recorded at each examination. Interpretation of diastolic indices was aligned, where feasible, with ASE/EACVI guideline-based principles, while acknowledging the practical constraints of immediate post-race field assessment.

Left ventricular ejection fraction (LVEF) was calculated using the Simpson biplane method of disc summation from apical four- and two-chamber views, in accordance with current ASE/EACVI guidelines, and averaged over three consecutive cardiac cycles in sinus rhythm. Post-race echocardiographic assessment was performed at the Cardiology Department of Giannitsa General Hospital, located approximately five minutes from the finish line by transport. Participants were placed in the left lateral decubitus position in a temperature-controlled examination room (~22 °C). Imaging commenced within 30 min of race completion. No fluid intake beyond customary post-race water provision was permitted between the finish line and the imaging suite. The same operator, equipment, and acquisition protocol were used for the pre- and post-race studies to minimise measurement variability. The LA area is reported here as a pragmatic field-based marker of acute atrial loading. Current ASE/EACVI guidelines recommend body-size-indexed LA volume (LAVI) as the preferred chamber-quantification metric for diastolic assessment; LAVI requires acquisition of orthogonal apical views with biplane disc summation, which was not consistently feasible in the immediate post-race setting given participant fatigue, breath-holding limitations, and time constraints. LA area, which has been shown to correlate strongly with LAVI in healthy populations, was therefore adopted as a feasible proxy. The LA area data presented should be interpreted as reflecting the acute trend observed in this exploratory study and not extrapolated to formal guideline-based diastolic classification.

Left ventricular diastolic indices included transmitral early (E) and late (A) filling velocities and their ratio (E/A), tissue Doppler early diastolic velocities at the lateral (e′ lat) and septal (e′ sept) annulus, average E/e′ ratio, left atrial (LA) anteroposterior diameter and area, and isovolumic relaxation time (IVRT). LA area was selected instead of LA volume index because it could be obtained rapidly and consistently in the immediate post-race setting; this choice improved feasibility but limited comparability with comprehensive guideline-based diastolic assessment. The use of LA area should therefore be interpreted as a pragmatic field-based marker of short-term atrial loading rather than a full substitute for indexed LA volume.

### 2.3. Biomarker Analysis

Venous blood samples were collected by standard venipuncture into serum separator tubes (SST). Samples were allowed to clot at room temperature, centrifuged at 3000× *g* for 10 min, and serum was analysed within 30 min of collection. Only one early post-race blood sample was obtained. Accordingly, the CRP value reflects only the earliest phase of the inflammatory response and cannot characterise the expected later peak at 24–72 h.

High-sensitivity cardiac troponin I (hs-cTnI) was measured using the Beckman Coulter Access hsTnI immunoassay (Beckman Coulter Inc., Brea, CA, USA). The limit of detection (LOD) was 0.34 ng/L, and the functional sensitivity (10% CV) was 1.35 ng/L. Sex-specific 99th percentile upper reference limits (URL) were 19.7 ng/L for males and 11.8 ng/L for females (0.0197 and 0.0118 ng/mL, respectively) [22]. Values exceeding the sex-specific URL were considered elevated. Serum CRP, total CPK, and CK-MB were analysed using standardised laboratory assays. Hydration status, body-mass change, plasma-volume change, and serial biomarker kinetics were not assessed and should be considered when interpreting the findings. These omissions are particularly important because both preload-related Doppler changes and biomarker concentrations may be modified by exercise-induced fluid shifts.

### 2.4. Statistical Analysis

Data were analysed using IBM SPSS Statistics Version 29.0.2.0 (IBM Corp., Armonk, NY, USA). Normality was assessed with the Shapiro–Wilk test. Pre/post comparisons were performed using paired t-tests or Wilcoxon signed-rank tests as appropriate. Correlations between change scores were assessed using Spearman’s rank correlation (Δ-biomarker vs. Δ-echocardiographic variables), given the non-normal distribution of most Δ-biomarker variables. Multiple linear regression was performed with a stepwise approach to identify preliminary correlates of ΔLA area and ΔE/A ratio; regression assumptions were verified using the Shapiro–Wilk test on residuals, visual inspection of residual plots, and Variance Inflation Factor scores. Effect sizes were calculated as Cohen’s d. Given the exploratory design, limited sample, and multiple endpoints, no formal correction for multiple comparisons was applied; *p*-values were interpreted cautiously, and emphasis was placed on overall patterns and effect-size magnitudes rather than isolated nominal significance. Results are presented as mean ± SD unless otherwise specified; for non-normally distributed variables, median [interquartile range] is reported alongside.

## 3. Results

### 3.1. Participant Characteristics

The cohort was predominantly male (80%; *n* = 16 males, *n* = 4 females). Twenty healthy amateur runners (mean age 50.7 ± 12.3 years; range 28–72 years) completed the race. Mean race completion time was 125 ± 8 min (Table 1).

### 3.2. Echocardiographic Findings

Post-race echocardiography demonstrated a coherent pattern of acute changes (Table 2). Three principal findings emerged. First, impaired diastolic relaxation: significant reductions in the E/A ratio and in both lateral and septal e′ velocities, accompanied by prolongation of IVRT (all *p* ≤ 0.003). Second, increased atrial loading: a significant rise in LA area (*p* < 0.001) together with a modest but significant increase in the average E/e′ ratio (*p* = 0.044). Third, a mild reduction in LVEF (*p* < 0.001) accompanied by a significant rise in heart rate (*p* < 0.001). Detailed values, 95% confidence intervals, and Cohen’s d effect sizes are reported in Table 2; the cardiac biomarker response is presented in Table 3. The parallel reduction in both lateral and septal e′ velocities suggests a diffuse rather than regional pattern of acute diastolic alteration. The biomarker elevations described below should be interpreted as reflecting the combined skeletal-muscle and myocardial stress of endurance exercise rather than acute myocardial injury (see Section 4).

### 3.3. Biomarker Response

Serum cardiac biomarkers exhibited significant post-race elevations (Table 3). CPK rose from 130.6 ± 104.8 to 522.4 ± 419.7 IU/L (*p* < 0.001), CK-MB from 9.95 ± 4.66 to 30.10 ± 14.05 ng/mL (*p* < 0.001), CRP from 0.061 ± 0.041 to 0.244 ± 0.165 mg/dL (*p* < 0.001), and hs-cTnI from 0.005 ± 0.001 to 0.025 ± 0.031 ng/mL (*p* = 0.013). Post-race hs-cTnI exceeded the sex-specific 99th percentile URL in 7 of 20 participants (35%; 4/16 males and 3/4 females). The maximum recorded post-race hs-cTnI value was 0.099 ng/mL, whereas no participant exceeded the sex-specific URL at baseline. All participants remained asymptomatic, and no new ECG changes were observed.

The pattern and magnitude of these biomarker elevations are consistent with the combined skeletal-muscle and myocardial stress of endurance exercise rather than with acute myocardial injury; the contextual interpretation, including the CK-MB/CPK ratio and the temporal characteristics of post-exercise hs-cTnI release, is addressed in Section 4.

### 3.4. Correlation Analysis

**Figure 1:**
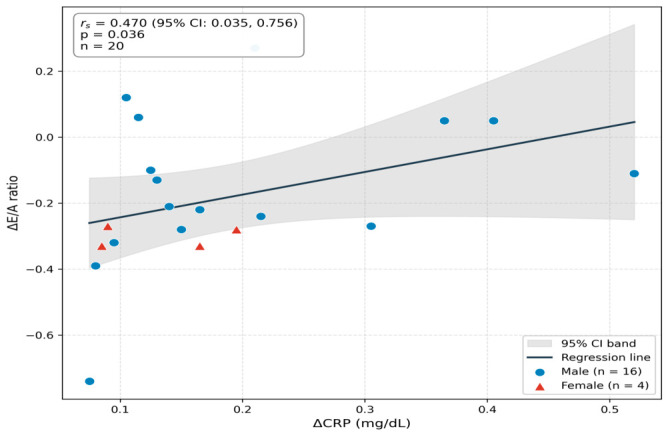
Correlation between ΔCRP and ΔE/A ratio (Spearman r_s_ = 0.470, *p* = 0.036, *n* = 20). Regression line with 95% CI band. Male participants shown as circles; female participants as triangles.

**Figure 2:**
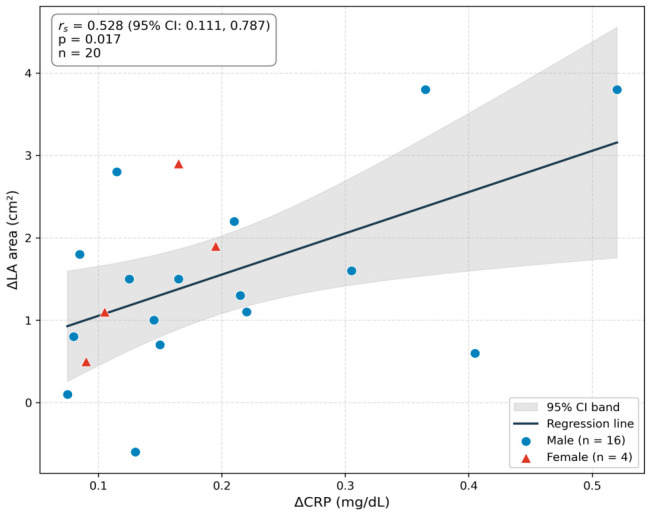
Correlation between ΔCRP and ΔLA area (Spearman r_s_ = 0.528, *p* = 0.017, *n* = 20).

**Figure 3:**
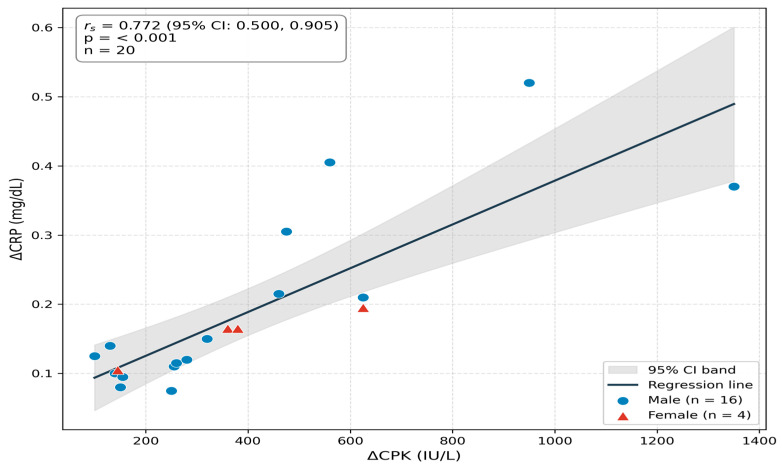
Correlation between ΔCPK and ΔCRP (Spearman r_s_ = 0.772, *p* < 0.001, *n* = 20).

**Figure 4:**
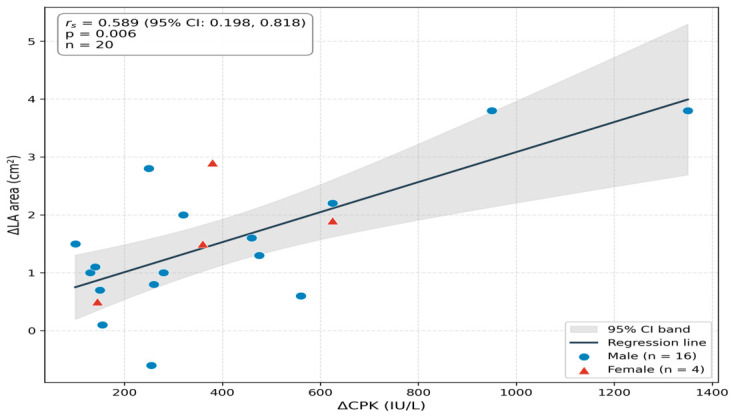
Correlation between ΔCPK and ΔLA area (Spearman r_s_ = 0.589, *p* = 0.006, *n* = 20).

**Figure 5:**
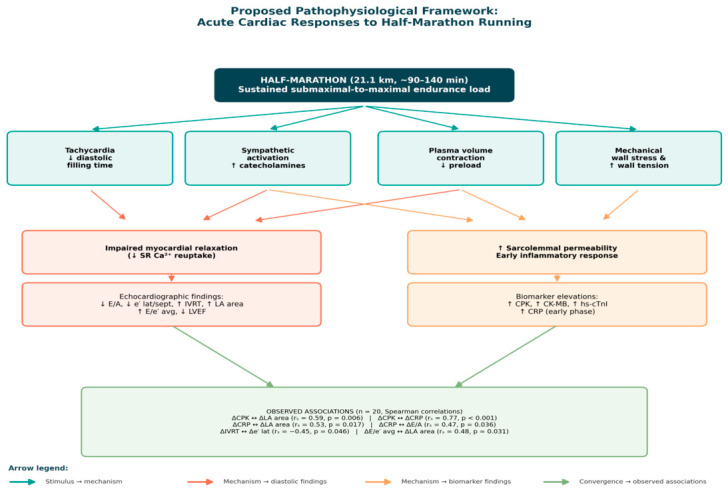
Proposed pathophysiological framework linking half-marathon running to the observed early cardiac responses. The diagram illustrates the principal acute mechanisms (tachycardia, sympathetic activation, plasma-volume contraction, mechanical wall stress) converging on two downstream pathways: impaired myocardial relaxation, manifesting as echocardiographic findings, and increased sarcolemmal permeability with early inflammatory response, manifesting as biomarker elevations. The observed Spearman correlations between these pathways are summarised at the base. Hydration status, plasma-volume change, and serial biomarker kinetics were not assessed; mechanistic links remain inferential. ↑, increase; ↓, decrease.

Exploratory Spearman rank correlations between Δ-echocardiographic and Δ-biomarker variables are summarised in Table 4. Several significant associations emerged: ΔCRP correlated positively with ΔE/A ratio (r_s_ = 0.470, *p* = 0.036) and with ΔLA area (r_s_ = 0.528, *p* = 0.017); ΔCPK correlated strongly with ΔCRP (r_s_ = 0.772, *p* < 0.001) and with ΔLA area (r_s_ = 0.589, *p* = 0.006); ΔCK-MB correlated with ΔCRP (r_s_ = 0.655, *p* = 0.002); ΔE/e′ average correlated with ΔLA area (r_s_ = 0.482, *p* = 0.031); and ΔIVRT correlated inversely with Δe′ lat (r_s_ = −0.451, *p* = 0.046). Selected scatter plots with regression lines and 95% confidence bands are provided in Figure 1, Figure 2, Figure 3 and Figure 4. All correlations are exploratory and should be interpreted as hypothesis-generating; no correction for multiple testing was applied (Figure 5).

### 3.5. Regression Analysis

Stepwise multiple linear regression was performed to identify preliminary correlates of ΔLA area and ΔE/A ratio (Table 5). Among candidate biomarker changes (ΔCPK, ΔCRP, ΔCK-MB, Δhs-cTnI), ΔCPK emerged as the principal correlate in both models. For ΔLA area, R^2^ = 0.495 (β = 0.703, *p* < 0.001), and for ΔE/A ratio, R^2^ = 0.263 (β = 0.513, *p* = 0.021). Regression assumptions were verified (Shapiro–Wilk test on residuals, visual inspection of residual plots, Variance Inflation Factor = 1.0 in both single-predictor models). It must be emphasised that stepwise variable selection with *n* = 20 is highly susceptible to capitalisation on chance; the reported R^2^ values are likely optimistic, and the regression findings should be regarded strictly as hypothesis-generating estimates requiring independent replication in adequately powered cohorts.

## 4. Discussion

In this exploratory study of amateur half-marathon runners, race completion was associated with early post-race changes in several echocardiographic diastolic indices together with increases in hs-cTnI, CK-MB, CPK, and CRP. The overall pattern is most appropriately interpreted as an acute snapshot of physiological cardiac stress rather than proof of established myocardial dysfunction or injury. From a cardiovascular health perspective, these findings are relevant because amateur athletes and their clinicians frequently face the challenge of interpreting early post-race abnormalities after strenuous exercise. The present design does not allow firm separation between transient loading-related changes, exercise-induced myocardial fatigue, and other contributors such as hydration status or biomarker kinetics [2,3,4,7,8,9].

Heart rate at the time of post-race echocardiography rose from a resting pre-race value of 58.0 ± 6.0 bpm to 75.0 ± 6.0 bpm. Tachycardia is a well-recognised rate-dependent confounder of Doppler-derived diastolic indices because shortening of the diastolic interval reduces the time available for early relaxation and passive filling. The post-race heart rate observed in our cohort, however, remained well below the range (approximately 90–100 bpm and above) at which rate-dependent compression of the diastolic interval is considered to critically distort filling. The observed reductions in E/A ratio, e′ velocities, and prolongation of IVRT are therefore unlikely to be attributable solely to tachycardia and more plausibly reflect a combination of early post-exercise myocardial relaxation perturbation and loading-related effects [3,4,8].

The echocardiographic findings should therefore be interpreted cautiously. Lower E/A ratio and e′ velocities together with longer IVRT are directionally consistent with prior reports of post-exercise diastolic perturbation. However, these indices are load- and rate-sensitive, and acute post-race reductions in preload or changes in venous return may influence them independently of intrinsic myocardial dysfunction. The concurrent increase in LA area and the post-race rise in heart rate support the possibility that altered loading conditions contributed to the observed pattern [2,3,4,10,23,24]. The persistence of changes in both lateral and septal e′ also favours a global physiological response over a focal regional process; however, this cannot be resolved definitively without deformation imaging.

The post-race heart rate increased significantly, from 58.0 ± 6.0 to 75.0 ± 6.0 bpm. Although this level is not extreme, heart rate remains a relevant confounder in the interpretation of Doppler-derived diastolic measurements because shortening of diastolic filling time can influence E-wave velocity, E/A ratio, and IVRT. Accordingly, the current data supports the presence of early post-race alterations in diastolic indices, but not a definitive diagnosis of intrinsic diastolic dysfunction [3,4].

Hydration-related effects are another important consideration. Dehydration and plasma-volume contraction after endurance exercise can reduce venous return and left ventricular end-diastolic volume, thereby lowering E-wave velocity and altering chamber dimensions. Because hydration status, body-mass change, and fluid intake were not measured, the relative contribution of preload reduction cannot be quantified in the present study. This is a major limitation and should temper mechanistic interpretation [23,24].

Interpretation of the post-race hs-cTnI elevations is also constrained by the absence of serial sampling. The kinetic profile of exercise-induced hs-cTnI release differs fundamentally from that of acute myocardial infarction: in athletes, hs-cTnI typically rises within 1–4 h of exercise cessation, peaks at 2–6 h, and returns to baseline within 24–48 h, whereas in acute myocardial injury, values continue to rise over the first 12–24 h [9,11,20]. A single 30-min post-race measurement, therefore, captures only the early ascending phase of this curve and cannot distinguish between the two patterns. The 35% URL exceedance rate observed here should be regarded as an early reading rather than a peak value, and serial sampling at 3–6, 12, 24, and 48 h would be required to formally characterise individual kinetic patterns and definitively differentiate physiological release from pathological injury.

A notable sex-related observation emerged from the individual hs-cTnI analysis. Three of four female participants (75%) exceeded the sex-specific 99th percentile URL post-race, compared with four of sixteen male participants (25%). Although the female subgroup is too small to support statistical inference, this disproportion warrants discussion. Plausible contributing mechanisms include lower baseline lean muscle mass and smaller cardiac chamber dimensions in women, which may translate into relatively greater myocardial wall stress for a given external workload; lower baseline hs-cTnI concentrations, which may render relative changes more likely to cross the URL; and described sex-related differences in haemodynamic and inflammatory responses to endurance exercise. Adequately powered, sex-balanced cohorts are required to determine whether female amateur half-marathon runners systematically exhibit higher rates of URL exceedance and, if so, what clinical implications this carries.

It is informative to position the half-marathon stimulus against the controlled environment of a clinical exercise test. A symptom-limited cardiopulmonary or treadmill exercise test typically lasts 8–12 min, peaks at ≥ 85% of the age-predicted maximum heart rate, and is performed under continuous medical supervision in a temperature-controlled setting with immediate access to resuscitation. A half-marathon, by contrast, imposes sustained submaximal-to-maximal load over approximately 90–140 min in variable environmental conditions, accompanied by cumulative dehydration, glycogen depletion, and progressive sympathetic activation. Both stimuli probe cardiac reserve, produce tachycardia and elevated stroke work, and can unmask subclinical disease in asymptomatic individuals; the half-marathon, however, exposes the heart to a far longer cumulative stress and reveals adaptations—particularly diastolic alterations and biomarker release—that a short clinical exercise test does not generate. The half-marathon may therefore be regarded as a real-world, prolonged, self-administered cardiac stress test, with the important caveat that it occurs without medical surveillance.

The breadth of our age range (28–72 years; mean 50.7 ± 12.3) warrants comment. Younger participants (28–40 years; *n* = 4) would be expected to have greater cardiovascular reserve, more complete training adaptation, and intrinsically better diastolic function at baseline, whereas older participants (≥60 years; *n* = 6) may show age-related reductions in early diastolic relaxation velocity and increased dependence on atrial contribution to filling. The pooled findings reported here, therefore, represent an averaged response across distinct physiological age strata. Qualitative inspection of individual data did not reveal clustering of abnormal post-race values within any single decade, but the sample size precludes formal age-stratified inferential analysis. Adequately powered cohorts with planned age stratification are required to determine whether older amateur runners exhibit qualitatively or quantitatively different acute cardiac responses to half-marathon competition.

The biomarker results also fit with the prior literature on endurance exercise. Increases in CPK and CK-MB are compatible with skeletal muscle stress and reduced biomarker specificity in the post-exercise setting, while the early rise in CRP should be understood as the onset of a systemic inflammatory response rather than its peak. The increase in hs-cTnI and the frequency of URL exceedance are clinically noteworthy, but a single early post-race sample cannot distinguish a benign exercise-related release pattern from a more persistent profile. This timing also creates an inherent interpretive mismatch between the early CRP signal and any later inflammatory burden that might be more biologically relevant to recovery. Serial measurements would be required to clarify peak timing and recovery [7,8,9,11,17,18].

CK-MB merits particular caution in interpretation because it is not myocardium-specific after prolonged exercise. In this context, the observed CK-MB increase should not be interpreted as evidence of myocardial injury in isolation. Rather, it should be considered alongside total CPK, symptoms, ECG findings, and the absence of serial imaging or biomarker data [8,9].

Post-race hs-cTnI exceeded the sex-specific 99th percentile URL in 35% of participants. A useful half-marathon-specific comparison is the recent study by Szałek-Goralewska et al. [20], which evaluated younger amateur runners and reported hs-cTnI elevations with less evident diastolic disturbance; the higher URL exceedance rate and clearer diastolic signal in our cohort may therefore relate, at least in part, to the older age and broader clinical profile of the present sample. However, without serial post-race sampling and follow-up imaging, the present study cannot determine whether these elevations represent only physiological exercise-related release or a more sustained myocardial response [9,11,20]. Accordingly, isolated early post-race hs-cTnI elevations in asymptomatic amateur runners should be interpreted alongside symptoms, ECG findings, and repeat testing rather than in isolation. Future studies should include sampling at 3–6 h, 24 h, and 48 h after the race, together with repeat imaging to define recovery kinetics more convincingly.

The decrease in LVEF after the race should likewise be interpreted conservatively. Post-exercise changes in loading conditions, end-diastolic volume, and heart rate can influence volumetric LVEF estimation, particularly when measurements are obtained immediately after endurance effort. In the absence of wall-motion abnormalities, symptoms, or follow-up studies, the observed reduction is best described as an early post-race change rather than definitive evidence of systolic impairment [23,24,25].

Recent longitudinal evidence further supports a cautious interpretation of acute post-exercise cardiac findings. Schindler et al. showed that transient post-marathon ventricular changes recovered within days and that exercise-induced troponin T release was not associated with long-term deterioration in ventricular function over 10 years in recreational runners [26]. While direct comparison with our cohort is limited by differences in race distance, biomarker assay, sex composition, and study design, these data reinforce the concept that acute biomarker elevation and early echocardiographic changes after endurance exercise do not necessarily imply persistent myocardial injury or maladaptive remodeling.

Taken together, these data support a practical message for cardiovascular health promotion: vigorous endurance exercise in apparently healthy amateur runners can be accompanied by transient early changes on echocardiography and biomarker testing. Awareness of this response pattern may improve post-exercise counselling, reduce over-interpretation of isolated early abnormalities, and help identify situations in which serial follow-up is appropriate. The study nevertheless has several important limitations: the small convenience sample, recruitment from a single running club, male predominance, absence of a non-exercising control group, no assessment of hydration before or after the race, use of LA area instead of LA volume index, lack of advanced imaging, and reliance on a single early post-race measurement. In addition, the pre-race assessment was performed within 30 days rather than immediately before the event. BMI and body surface area were not collected, preventing indexed chamber interpretation, and no correction for multiple testing was applied. These limitations mean that the present findings should be interpreted as exploratory and should not be used to infer definitive myocardial dysfunction or injury.

Several additional limitations warrant explicit acknowledgement. First, the use of conventional Doppler and tissue Doppler indices without speckle-tracking-derived global longitudinal strain (GLS) reduces sensitivity to subclinical myocardial dysfunction; GLS is recommended in current ASE/EACVI guidelines for the evaluation of subtle myocardial deformation, and its absence here reflects the constraints of the acute post-race field setting and the equipment available. Second, the broad age range (28–72 years), coupled with the small sample size, precludes formal age-stratified analysis. Third, the multiplicity of pre/post-comparisons in an exploratory design without formal correction for multiple testing increases the probability of nominally significant findings arising by chance; for this reason, effect sizes (Cohen’s d) are reported prominently alongside *p*-values throughout, and 95% confidence intervals have been added to Table 2 and Table 3 to allow readers to weigh both magnitude and precision. Fourth, the predominantly male composition of the cohort (80%) limits generalisability to female athletes and precludes sex-stratified inferential analysis. Fifth, quantitative training-load monitoring during the 30-day pre-race window was not performed. Sixth, formal race-day environmental monitoring (temperature, humidity, wind) was not part of the protocol. The hypothesis-generating regression findings (Section 3.5) carry a high risk of overfitting at *n* = 20 with stepwise selection and require replication.

## 5. Conclusions

In amateur runners, half-marathon completion was associated with early post-race changes in left ventricular diastolic indices and increases in hs-cTnI, CPK, CK-MB, and CRP. The findings are consistent with acute physiological cardiac stress; subclinical myocardial injury cannot be excluded without serial biomarker assessment and advanced imaging. Within the context of exercise-based cardiovascular health promotion, these data may help contextualise early post-race findings and support safer participation and clinical follow-up strategies after vigorous endurance exercise. Larger prospective studies with sex-balanced recruitment, hydration assessment, and serial follow-up at 3–6 h, 24 h, and 48 h are needed to clarify clinical significance and recovery patterns.

## Figures and Tables

**Table 1:** Demographic and training characteristics of the study cohort (*n* = 20).

Variable	Mean ± SD	Median	Range
Age (years)	50.7 ± 12.3	49.5	28–72
Experience in endurance events (years)	11.35 ± 3.8	10	7–17
Weekly training hours	7.0 ± 1.4	7.0	5.6–9.6
Races per year	5.85 ± 1.2	6.0	4–8
Sex	M 80% (*n* = 16); F 20% (*n* = 4)	—	—
Half-marathon time (min)	125 ± 8	127	93–140

**Table 2:** Echocardiographic parameters pre- and post-half-marathon (*n* = 20).

Parameter	Pre-Race	Post-Race	Δ Mean ± SD	*p*-Value	95% CI	Cohen’s d
Heart rate (bpm)	58.0 ± 6.0	75.0 ± 6.0	+17.0 ± 1.6	<0.001 a	(16.25, 17.75)	— *
LVEF (%)	65.0 ± 6.5	58.8 ± 5.9	−6.2 ± 5.1	<0.001 b	(−8.59, −3.81)	1.22
RV EDD (cm)	3.60 ± 0.61	3.86 ± 0.55	+0.27 ± 0.28	<0.001 a	(0.14, 0.40)	0.96
TAPSE (cm)	2.54 ± 0.42	2.45 ± 0.45	−0.08 ± 0.28	0.417 a	(−0.21, 0.05)	0.29
E (m/s)	0.74 ± 0.17	0.69 ± 0.17	−0.05 ± 0.14	0.175 a	(−0.12, 0.02)	0.36
A (m/s)	0.57 ± 0.12	0.62 ± 0.13	+0.06 ± 0.10	0.021 a	(0.01, 0.11)	0.60
E/A ratio	1.35 ± 0.36	1.16 ± 0.37	−0.19 ± 0.22	<0.001 a	(−0.29, −0.09)	0.86
e′ lat (m/s)	0.154 ± 0.027	0.135 ± 0.020	−0.019 ± 0.020	0.003 b	(−0.028, −0.010)	0.95
e′ sept (m/s)	0.133 ± 0.027	0.110 ± 0.024	−0.022 ± 0.018	<0.001 a	(−0.030, −0.014)	1.22
E/e′ avg	5.31 ± 1.38	5.83 ± 1.50	+0.52 ± 1.56	0.044 b	(−0.21, 1.25)	0.33
LA area (cm^2^)	15.55 ± 2.81	17.10 ± 3.26	+1.55 ± 1.15	<0.001 a	(1.01, 2.09)	1.35
IVRT (ms)	74.4 ± 12.9	89.0 ± 13.7	+14.6 ± 9.8	<0.001 a	(10.01, 19.19)	1.49

a—paired *t*-test; b—Wilcoxon signed-rank test. — * = Cohen’s d not reported (very low SD of differences for heart rate reflects uniform tachycardic response).

**Table 3:** Cardiac biomarkers pre- and post-half-marathon (*n* = 20). hs-cTnI reference limits: Beckman Coulter Access hsTnI sex-specific 99th percentile URL [22]. All p-values from Wilcoxon signed-rank tests (Δ values non-normally distributed by Shapiro–Wilk).

Biomarker	Pre-Race Mean ± SD Median [IQR]	Post-Race Mean ± SD Median [IQR]	Δ Mean ± SD	*p* (Reference Range)	95% CI	Cohen’s d
CRP (mg/dL)	0.061 ± 0.041 0.045 [0.038]	0.244 ± 0.165 0.180 [0.153]	+0.183 ± 0.124	<0.001 (<0.5 mg/dL)	(0.125, 0.241)	1.48
CPK (IU/L)	130.6 ± 104.8 99.5 [115.0]	522.4 ± 419.7 398.0 [461.3]	+391.8 ± 314.8	<0.001 (M: 30–200; F: 29–168)	(244.5, 539.1)	1.24
CK-MB (ng/mL)	9.95 ± 4.66 8.50 [6.50]	30.10 ± 14.05 25.50 [19.25]	+20.15 ± 9.40	<0.001 (<5.0 ng/mL)	(15.75, 24.55)	2.14
hs-cTnI (ng/mL)	0.005 ± 0.001 0.005 [0.002]	0.025 ± 0.031 0.009 [0.033]	+0.020 ± 0.031	0.013 (M: <0.0197; F: <0.0118)	(0.0055, 0.0345)	0.65

**Table 4:** Spearman rank correlations between Δ-echocardiographic and Δ-biomarker variables (*n* = 20). 95% CIs derived via Fisher z-transformation. All correlations are exploratory.

Variable Pair	Spearman r_s_	95% CI	*p*-Value
ΔCRP vs. ΔE/A ratio	0.470	(0.035, 0.756)	0.036
ΔCRP vs. ΔLA area	0.528	(0.111, 0.787)	0.017
ΔCPK vs. ΔCRP	0.772	(0.500, 0.905)	<0.001
ΔCPK vs. ΔLA area	0.589	(0.198, 0.818)	0.006
ΔCK-MB vs. ΔCRP	0.655	(0.301, 0.852)	0.002
ΔE/e′ avg vs. ΔLA area	0.482	(0.052, 0.762)	0.031
ΔIVRT vs. Δe′ lat	−0.451	(−0.745, −0.013)	0.046

**Table 5:** Stepwise multiple linear regression with ΔCPK as predictor (*n* = 20). β = standardised regression coefficient; VIF = Variance Inflation Factor. Findings are exploratory and susceptible to overfitting at this sample size.

Outcome	Predictor	β	R^2^	*p*-Value	VIF
ΔLA area	ΔCPK	0.703	0.495	<0.001	1.0
ΔE/A ratio	ΔCPK	0.513	0.263	0.021	1.0

## Data Availability

The data presented in this study are available from the corresponding author upon reasonable request, subject to ethical and institutional restrictions.

## Abbreviations

The following abbreviations are used in this manuscript:

CRP	C-reactive protein
CPK	creatine phosphokinase
CK-MB	creatine kinase-myocardial band
E/A	transmitral early/late filling ratio
hs-cTnI	high-sensitivity cardiac troponin I
IVRT	isovolumic relaxation time
LA	left atrium
LVEF	left ventricular ejection fraction
